# AI-Based Facial Phenotyping Supports a Shared Molecular Axis in *PACS1*-, *PACS2*-, and *WDR37*-Related Syndromes

**DOI:** 10.3390/ijms26167964

**Published:** 2025-08-18

**Authors:** Julia del Rincón, Marta Gil-Salvador, Cristina Lucia-Campos, Laura Acero, Laura Trujillano, María Arnedo, Pilar Pamplona, Ariadna Ayerza-Casas, Beatriz Puisac, Feliciano J. Ramos, Juan Pié, Ana Latorre-Pellicer

**Affiliations:** 1 Unit of Clinical Genetics and Functional Genomics, Department of Pharmacology and Physiology, School of Medicine, University of Zaragoza, CIBERER-GCV2 and IIS-Aragon-GIIS062, 50009 Zaragoza, Spainmarnedo@unizar.es (M.A.); puisac@unizar.es (B.P.); juanpie@unizar.es (J.P.); 2 Clinical and Molecular Genetics Area, Vall d’Hebron Hospital, Medicine Genetics Group, Vall d’Hebron Research Institute (VHIR), 08035 Barcelona, Spain; lautrujillano@gmail.com; 3 Unit of Neurophysiology, University Hospital Lozano Blesa, 50009 Zaragoza, Spain; 4 Unit of Paediatric Cardiology, Service of Paediatrics, University Hospital Miguel Servet, 50009 Zaragoza, Spain; 5 Unit of Clinical Genetics, Service of Paediatrics, Department of Paediatrics, University Hospital Lozano Blesa, School of Medicine, University of Zaragoza, CIBERER-GCV2 and IIS-Aragon-GIIS062, 50009 Zaragoza, Spain

**Keywords:** Face2Gene, AI-based facial analysis, dysmorphology, gestalt, rare genetic diseases, Schuurs-Hoeijmakers syndrome, SHMS, *PACS1*, *PACS2*, *WDR37*

## Abstract

Despite significant advances in gene discovery, the molecular basis of many rare genetic disorders remains poorly understood. The concept of disease modules, clusters of functionally related genes whose disruption leads to overlapping phenotypes, offers a valuable framework for interpreting these conditions. However, identifying such relationships remains particularly challenging in ultra-rare syndromes due to the limited number of documented cases. We hypothesized that AI-based facial phenotyping could aid in identifying shared molecular mechanisms by detecting phenotypic convergence among clinically related syndromes. To test this, we used Schuurs–Hoeijmakers syndrome (SHMS; OMIM #615009), caused by a recurrent de novo variant in *PACS1*, as a model to explore potential phenotypic and functional associations with *PACS2*-related disorder (DEE66; OMIM #618067) and *WDR37*-related disorder (NOCGUS; OMIM #618652). Facial photographs of individuals with SHMS were analyzed using the DeepGestalt and GestaltMatcher algorithms. In addition to consistently recognizing SHMS as a distinct clinical entity, the algorithms frequently matched DEE66 and NOCGUS, suggesting a shared facial gestalt. Binary comparisons further confirmed overlapping craniofacial features among the three disorders. These findings were supported by literature review, indicating clinical overlapping and potential functional associations. Overall, our results confirm the presence of consistent facial similarities among *PACS1*-, *PACS2*-, and *WDR37*-related syndromes and highlight the utility of AI-driven facial phenotyping as a complementary tool for uncovering clinically relevant relationships in ultra-rare genetic disorders.

## 1. Introduction

Rare genetic disorders (RGDs) affect an estimated 3.5–5.9% of the global population [1], with more than 7000 conditions currently described and nearly 5000 genes linked to phenotype-causing mutations [2]. The increasing use of high-throughput sequencing technologies, along with international research collaborations, has greatly accelerated the discovery of novel disease-associated genes over the past decade. Despite these advances, understanding the underlying biological mechanisms of RGDs remains a major challenge, particularly in the context of ultra-rare conditions [3,4,5].

More than 80% of RGDs manifest during childhood and frequently affect the central nervous system (CNS), with neurological symptoms reported in over 70% of cases [6,7,8]. Despite their marked genetic and clinical heterogeneity, growing evidence from network-based studies indicates that disease-associated genes often cluster within interconnected biological pathways, such as synaptic transmission, chromatin remodeling, or mTOR signaling, rather than acting in isolation [9,10]. These findings support the concept of disease modules: groups of functionally related genes whose disruption results in overlapping phenotypes [11].

Identifying such relationships in ultra-rare disorders is particularly difficult due to the small number of reported cases. However, phenotypic similarities, especially those involving craniofacial features, can provide important diagnostic and biological clues. Many RGDs, particularly NDDs, exhibit recurrent craniofacial patterns or a recognizable “gestalt” that can guide clinical and molecular hypothesis generation. The emergence of artificial intelligence (AI)-based facial phenotyping tools has transformed this process [12,13,14]. Algorithms such as DeepGestalt and GestaltMatcher, implemented in platforms like Face2Gene, use deep learning to detect and compare facial features, suggesting candidate syndromes or phenotypically similar conditions based on a patient’s facial image [15,16,17].

In addition to their diagnostic value, AI-based facial phenotyping tools have been proposed as a means to uncover biologically meaningful relationships among rare disorders [18]. In this study, we explore that potential by analyzing individuals with Schuurs–Hoeijmakers syndrome (SHMS, OMIM #615009), also known as PACS1 syndrome, a rare autosomal dominant NDD caused by a recurrent de novo variant in the *PACS1* gene (c.607C>T, p.Arg203Trp) [19]. *PACS1* encodes a multifunctional protein implicated in intracellular trafficking [20], chromatin regulation [21], and neural development [22]. However, the precise pathophysiological consequences of this variant remain poorly understood [23]. Notably, previous studies have identified clinical and molecular similarities between SHMS and disorders associated with variants in *PACS2* (DEE66; OMIM #618067) and *WDR37* (NOCGUS; OMIM #618652) [24,25,26], suggesting the existence of a PACS1–PACS2–WDR37 axis that may underlie shared phenotypic features across their respective syndromes.

Building on these observations, the aim of this study is to assess whether AI-driven facial analysis can detect phenotypic convergence among *PACS1*-, *PACS2*-, and *WDR37*-related syndromes, and whether such resemblance aligns with known or hypothesized functional relationships. While facial similarity patterns can be informative, they must be interpreted with caution and supported by additional biological evidence. To this end, we complemented our facial analysis with structured phenotypic annotation using the Human Phenotype Ontology (HPO) and a comprehensive review of the relevant literature. This integrative approach allows for a more robust interpretation of phenotypic convergence and supports the use of AI-driven phenotyping as a complementary strategy to identify biologically meaningful relationships among RGDs.

## 2. Results

### 2.1. Dysmorphic Facial Phenotype in Individuals with Schuurs–Hoeijmakers Syndrome

**Figure 1:**
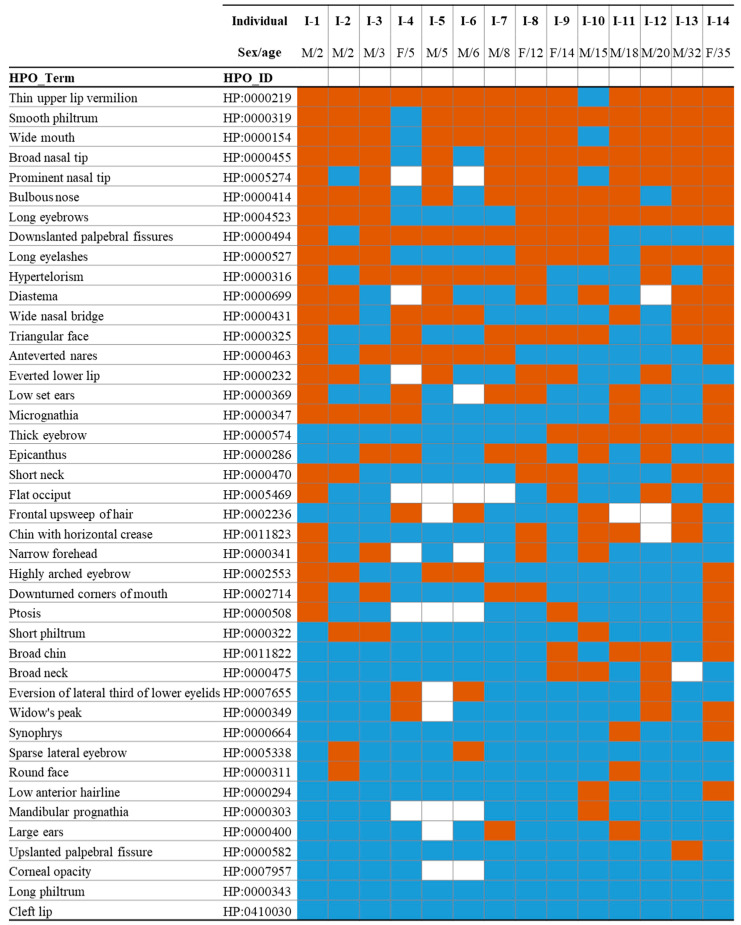
Craniofacial clinical evaluation of individuals with Schuurs–Hoeijmakers syndrome (SHMS). Feature presence is indicated in orange, absence in blue, and unknown or not applicable status in white, based on Human Phenotype Ontology (HPO) terms. M, male; F, female.

We report on 14 patients with the genetic variant c.607C>T, p.(Arg203Trp) in the *PACS1* gene. The cohort comprised four females and ten males, aged between 2 and 35 years. A clinical summary of their facial features is presented in Figure 1. Overall, the most common craniofacial features observed included thin upper lip vermilion (HP:0000219), smooth philtrum (HP:0000319), wide mouth (HP:0000154), broad and prominent nasal tip (HP:0000455, HP:0005274), and bulbous nose (HP:0000414) (Figure 1).

Facial photographs of the 14 individuals were analyzed using the Face2Gene CLINIC application with the DeepGestalt algorithm (v.22.3.0). These photos had not been used in any prior training of the algorithm. SHMS was recommended among the top 30 syndromes and ranked as the first diagnosis for 42.86% (*n* = 6) of individuals, second for 14.26% (*n* = 2), and third for 21.43% (*n* = 3). Overall, 92.86% (*n* = 13) of the patient photos analyzed had SHMS ranked in their top ten potential diagnoses out of the 30 possible suggested syndromes, from among the more than 350 syndromes currently recognized by the DeepGestalt algorithm. Among these 13 with SHMS in the top ten rank, the median gestalt score was 0.30 ± 0.16 (Table S1).

**Figure 2:**
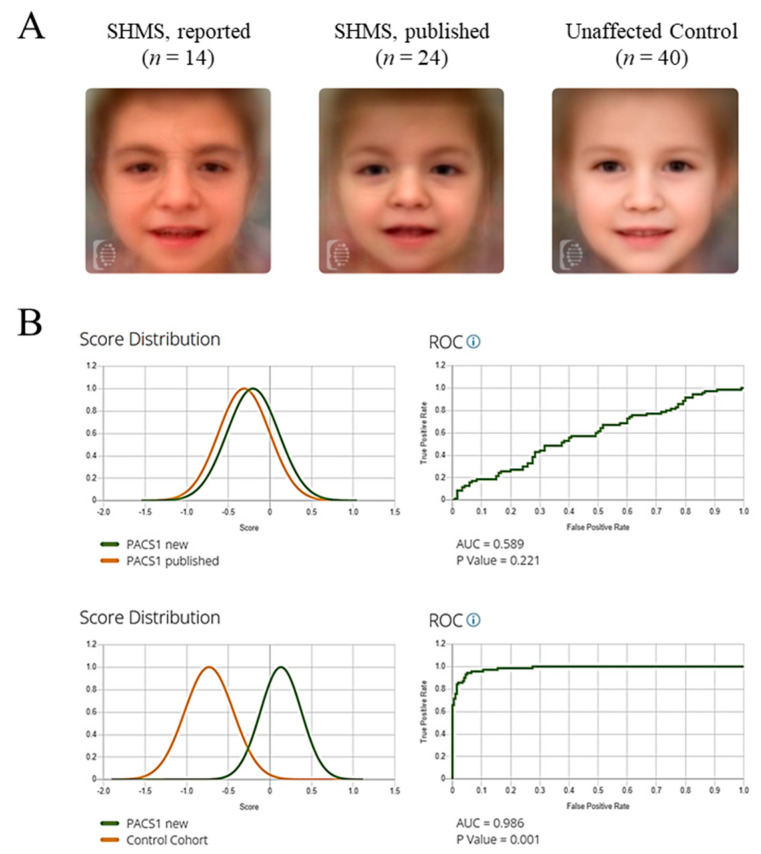
Binary comparison of facial images of individuals with SHMS and unaffected controls. (**A**) Composite gestalt based upon 14 participants with SHMS, 24 previously published individuals with SHMS, and 40 unaffected individuals. (**B**) Score distribution and ROC curve obtained using DeepGestalt analysis. Upper panels: comparison between newly reported SHMS individuals and previously published cases. Lower panels: comparison between newly reported SHMS individuals and unaffected controls.

To evaluate facial similarity between our cohort (*n* = 14) and previously published cases of SHMS (*n* = 24) (Table S2) [19,27,28,29,30,31,32], we used binary comparison in the Face2Gene RESEARCH (v.22.3.0) application. Model performance was assessed using Receiver Operating Characteristic (ROC) curves, with the Area Under the Curve (AUC) reflecting discriminatory power. An AUC close to 1.0 indicates strong performance; values near 0.5 suggest random classification. The comparison yielded an AUC of 0.589 (*p* = 0.221), indicating no significant difference between the two SHMS groups. In contrast, a comparison between unaffected individuals (*n* = 40) and SHMS patients yielded an AUC of 0.986 with statistical significance (*p* < 0.001), confirming that SHMS presents a reasonably distinguishable facial gestalt (Figure 2). It is important to note that factors such as patient age, sex, and photo quality may influence the performance of the DeepGestalt algorithm. While this study did not systematically analyze these variables, the consistent identification of SHMS across varied demographics suggests a robust performance.

### 2.2. Common Differential Syndromes of SHMS Based on Gestalt Analysis

Based on the results above, photographs of the 14 individuals with SHMS from the present cohort and the 24 from previous publications were analyzed as a single group (*n* = 38) using DeepGestalt and GestaltMatcher (v.1.2.0) algorithms. We inquired about the common syndromes suggested in more than 50% of the photographs analyzed.

First, we applied the DeepGestalt algorithm. In addition to SHMS, another 10 syndromes were suggested among the 30 top syndrome matches (Table 1). Next, we examined ultra-rare syndromes using the GestaltMatcher algorithm. Curiously, developmental and epileptic encephalopathy-66 (DEE66; OMIM #618067), caused by alterations in *PACS2* gene, was suggested in 33 of the 38 patients analyzed. The combination of both algorithms, DeepGestalt and GestaltMatcher, is graphically displayed as a tSNE plot in the Face2Gene CLINIC app. Using this application, the top syndromes matched by both algorithms were SHMS (37/38); DEE66 (30/38); Verheij syndrome (VRJS, OMIM #615583) (26/38), caused by a gene deletion involving the *PUF60* gene; neuro-oculo-cardio-genitourinary syndrome (NOCGUS, OMIM #618652) (23/38), caused by heterozygous variants in the *WDR37* gene; and Baraitser–Winter syndrome 1 (BRWS1, OMIM #243310) (20/38), caused by alterations in the *ACTB* gene (Table 1). Interestingly, the two syndromes previously proposed to share a molecular basis with PACS1, DEE66, and NOCGUS, emerged prominently in this analysis.

### 2.3. Quantification of Facial Gestalt Similarities Between Syndromes

Both DeepGestalt and GestaltMatcher quantify facial similarities using deep convolutional neural networks. We applied these tools to assess potential phenotypic overlaps that might indicate shared molecular pathways. In addition to individuals with SHMS (*n* = 38), we included publicly available facial images from academic publications of patients with DEE66 (*n* = 11) [25,33,34,35,36,37], VRJS (*n* = 23) [38,39,40,41], NOCGUS (*n* = 10) [42,43,44], and BRWS1 *(n* = 32) [45,46,47,48,49,50] (Table S3). These syndromes were prioritized for binary comparisons due to their consistent co-suggestion by the AI-based algorithms.

**Figure 3:**
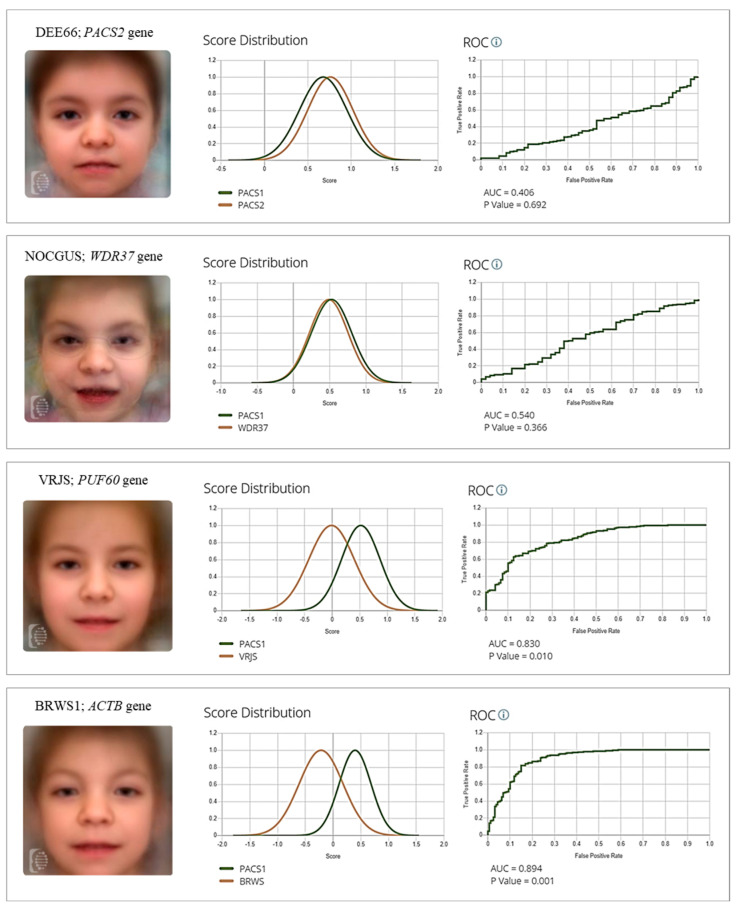
Binary comparison of facial images of individuals with SHMS (*n* = 38) versus Epileptic Encephalopathy, Early Infantile, 66 (DEE66) (*n* = 11), Neuro-Oculo-Cardio-Genitourinary syndrome (NOCGUS) (*n* = 10), Verheij syndrome (VRJS) (*n* = 23), and Baraitser–Winter syndrome (BRWS1) (*n* = 32). **Left**: Composite gestalt image obtained based on the analyzed photographs. **Right**: Score distribution and ROC curve obtained by DeepGestalt analysis.

Binary comparisons revealed no statistically significant differences between SHMS and DEE66 (AUC = 0.406; *p* = 0.692), SHMS and NOCGUS (AUC = 0.540; *p* = 0.366), or DEE66 and NOCGUS (AUC = 0.493; *p* = 0.458), suggesting potential facial overlap among these *PACS1*-, *PACS2*-, and *WDR37*-related syndromes and potential molecular convergence. In contrast, the algorithm was able to differentiate SHMS from VRJS (AUC = 0.830; *p* = 0.010) and BRWS1 (AUC = 0.894; *p* = 0.001), supporting the distinctiveness of the SHMS facial phenotype in these comparisons (Figure 3).

Given the historical diagnostic overlap between SHMS and Cornelia de Lange syndrome (CdLS), and the fact that CdLS appeared among the top matches in over 50% of SHMS cases (Table 1), we also assessed this comparison. SHMS and CdLS were clearly differentiated (AUC = 0.996; *p* < 0.001), further reinforcing the specificity of the SHMS facial phenotype (Figure S1).

Taken together, these findings suggest that *PACS1*-, *PACS2*-, and *WDR37*-related syndromes share a recognizable facial signature that may reflect a common biological basis.

## 3. Discussion

Many rare genetic disorders (RGDs) are characterized by distinctive craniofacial features, making dysmorphological assessment a long-standing cornerstone of clinical genetics. In recent years, the integration of Human Phenotype Ontology (HPO) terminology and AI-based image analysis tools has significantly advanced the field, enabling the extraction of quantitative phenotypic descriptors from facial photographs. These technologies have enhanced diagnostic accuracy and supported clinical decision-making in genetics [51,52].

In this context, the application of AI-driven facial phenotyping in our study provides new perspectives on phenotypic and functional convergence among RGDs with overlapping facial features. Using Schuurs–Hoeijmakers syndrome (SHMS, or PACS1 syndrome) as a reference model, we demonstrate how computational analysis can refine syndrome-specific facial profiling and reveal phenotypic relationships across genetically unrelated conditions. The consistent identification of PACS1 syndrome by both DeepGestalt and GestaltMatcher supports the existence of a distinctive and well-characterized facial gestalt, reinforcing its classification as a distinct clinical entity [27,28,29,53,54]. In our cohort, DeepGestalt correctly ranked SHMS within the top 10 diagnoses in 92.86% of cases, an outcome consistent with its reported performance across other RGDs [16,17,55]. Beyond their diagnostic potential, the frequent co-identification of SHMS with Developmental and Epileptic Encephalopathy 66 (DEE66, linked to *PACS2* variants) and Neuro-Oculo-Cardio-Genitourinary syndrome (NOCGUS, caused by *WDR37* variants) suggests notable facial similarity despite distinct molecular etiologies. The low AUC values observed in binary comparisons among these syndromes indicate a high degree of morphological resemblance, demonstrating the sensitivity of AI-based approaches in detecting subtle phenotypic convergence. Importantly, these tools also preserved their discriminatory power, as evidenced by their ability to distinguish SHMS from Cornelia de Lange syndrome (CdLS), a historically difficult differential diagnosis. Taken together, these findings emphasizes the dual utility of AI-driven facial analysis, both as a diagnostic aid and as a hypothesis-generating tool for exploring shared developmental mechanisms in ultra-rare syndromes.

Nevertheless, our findings should be interpreted with caution. The limited ability of the model to distinguish SHMS, DEE66, and NOCGUS may reflect genuine phenotypic convergence, but could also result from technical constraints or small sample sizes, particularly for DEE66 and NOCGUS, which may affect generalizability and increase the risk of overfitting. These limitations must be considered when inferring shared molecular mechanisms from AI-derived phenotypic similarities. Looking ahead, emerging multimodal AI systems, including those incorporating large language models, may further improve diagnostic precision by integrating facial, clinical, genetic, and textual data [56,57].

From a clinical standpoint, syndromes associated with *PACS1*, *PACS2*, and *WDR37* display marked phenotypic overlap. All three present with facial dysmorphism, significant neurodevelopmental impairment, and a wide range of neurological features [23]. Although *PACS2*-related syndrome primarily affects the nervous system, it also presents with clinical signs common to PACS1 and WDR37 syndromes, including feeding difficulties, postnatal growth retardation, and variable skeletal, cardiac, and genitourinary anomalies [56]. Ocular abnormalities like corneal opacity and coloboma are more frequent in WDR37 cases but have also been reported with *PACS1* and *PACS2* variants [24]. Seizures are a prominent hallmark across all three syndromes, particularly severe and often refractory in PACS2 and WDR37, with onset typically in early infancy [56]. Cerebellar hypoplasia and other brain abnormalities are frequently observed, especially in *WDR37*-related presentations, which also tend to show more profound neurocognitive impairment and multisystem involvement, including rare cases of early mortality [24,42]. In terms of craniofacial gestalt, common features include hypertelorism, broad nasal bridge, bulbous nose, short and smooth philtrum, thin upper lip, and wide mouth. While some traits such as arched eyebrows, sparse lateral eyebrows, and prominent pinnae appear more frequently in *PACS2*- and *WDR37*-related cases, these differences are subtle and not exclusive. Mild distinctions can be noted; triangular facial shape tends to be more frequently reported in PACS1, microcephaly, craniosynostosis, and auricular anomalies in WDR37, and synophrys or the absence of a smooth philtrum in a subset of PACS2 cases.

This significant phenotypic overlap supports the hypothesis that *PACS1*, *PACS2*, and *WDR37* may participate in a shared molecular pathway involved in neurodevelopment and craniofacial morphogenesis. Transcriptomic data show that all three genes are expressed during early embryogenesis, particularly in neural crest-derived tissues relevant to facial development. *PACS1*, for instance, demonstrates high expression in the developing brain and facial mesenchyme, suggesting its role in early craniofacial patterning [58]. This aligns with current models of neurodevelopmental disorders, which emphasize the coordinated regulation of craniofacial and brain development by neural crest gene regulatory networks, and how their disruption can result in overlapping syndromic features [59].

At the molecular level, PACS1 and PACS2 are evolutionarily conserved paralogs that regulate intracellular trafficking by interacting with acidic cluster motifs via their furin-binding region (FBR). They are involved in the localization of key cytoplasmic proteins such as receptors, proteases, and ion channels [60,61,62,63], and also engage in nuclear processes related to chromatin regulation and DNA repair through interactions with class I and III histone deacetylases (HDACs) [21,64]. Beyond their well-established role in protein trafficking, PACS proteins have been identified as interaction partners of WDR37, a conserved WD40 repeat-containing protein, across multiple species ranging from invertebrates to mammals. The interaction between PACS1 and WDR37 has been shown to influence endoplasmic reticulum (ER) calcium signaling [25,65]. This interaction supports their functional interdependence and provides strong evidence that PACS1, PACS2, and WDR37 constitute a conserved molecular axis.

In summary, this study highlights the potential of combining AI-driven facial phenotyping with molecular and clinical data to uncover biologically meaningful relationships in RGDs. Our findings suggest that *PACS1*-, *PACS2*-, and *WDR37*-associated syndromes form a coherent phenotypic and molecular cluster. While further validation is necessary, this integrative approach offers a scalable framework to identify hidden connections among RGDs, with potential implications for diagnosis, disease classification, and the understanding of potential biological links among ultra-rare syndromes.

## 4. Materials and Methods

### 4.1. Patient Recruitment and Clinical Imaging

Clinical and genotyping information from 14 unrelated individuals diagnosed with PACS1 syndrome was obtained. Participants were recruited through a call for collaboration by the Spanish PACS1 Patient Association. Inclusion criteria comprised a confirmed clinical and molecular diagnosis of SHMS, availability of a detailed clinical evaluation, and the presence of a frontal facial photograph. Informed consent was obtained from parents or guardians of all individuals included in this study, in accordance with the local ethics committee (CEICA; PI15/00707, PI24/288). Written informed consent for the publication of photographs was also obtained. Facial images were uploaded to the Face2Gene CLINIC App for phenotypic analysis. Additional photographs used in this work were obtained from scientific literature, as appropriately referenced in each section. All selected images were standardly cropped, centered, and aligned. Control photographs, matched for age, sex, and ethnicity, were supplied by Face2Gene technical support. Photographs of individuals with Cornelia de Lange syndrome (CdLS) were obtained under the same ethics approval (CEICA; PI15/00707, PI24/288) and were collected through collaboration with the Spanish Cornelia de Lange Association.

### 4.2. Clinical Facial Deep Phenotyping

Phenotypic characterization of patients was performed by a US Board-certified clinical geneticist during clinical evaluation. Clinical data were collected using a standardized restricted-term questionnaire, and detailed phenotypic descriptions of the individuals were annotated using the Human Phenotype Ontology (HPO) nomenclature (https://hpo.jax.org/, accessed on 3 March 2025). A total of 42 HPO terms related to craniofacial features were included in the questionnaire.

### 4.3. Computational Facial Analysis

Computational facial analysis was performed by Face2Gene (F2G) applications (FDNA Inc., Boston, MA, USA) using three algorithms: DeepGestalt (v.22.3.0), GestalMatcher (v.1.2.0) and the newly described facial D-score (v.2.0). The DeepGestalt algorithm first preprocesses each photo to detect facial landmarks and align the face, which is then cropped into facial regions. Each region is fed into a Deep Convolutional Neural Network (DCNN) to generate a descriptor and a softmax vector indicating its correspondence to each syndrome included in the model. The output vectors of all regional DCNNs are then aggregated and sorted to produce the final ranked list of 30 suggested genetic syndromes displayed in Face2Gene, each assigned a gestalt score, where higher values reflect greater similarity in facial morphology to a particular disorder [16]. GestaltMatcher uses these descriptors to create a “Clinical Face Phenotype Space”, in which the distance between photos defines syndromic similarity, calculated via cosine distance in that space and expressed as a “similarity score” [19]. The facial D-score algorithm uses the same descriptors to distinguish between two classes of frontal facial photos: images of patients diagnosed with a rare genetic disease showing facial dysmorphic features, and an equivalently sampled control group of unaffected individuals. Scores above 0.75 indicate a likely presence of facial dysmorphia [35].

## Figures and Tables

**Table 1:** Syndromes identified by the DeepGestalt and GestaltMatcher algorithms in more than 50% of individuals with SHMS analyzed (*n* = 38).

Syndrome_id	OMIM_id	Gene	# Patients	Gestalt_Score (Prom)
DeepGestalt				
SHMS	615009	*PACS1*	37	0.558
BRWS1	243310	*ACTB*	33	0.244
NS1	163950	*PTPN11*	33	0.297
KABUK1	147920	*KMT2D*	28	0.223
RSTS1	180849	*CREBBP*	25	0.213
AS	105830	*UBE3A*	24	0.226
NF1	162200	*NF1*	24	0.219
CdLS	122470	*NIPBL*	23	0.215
CSS	135900	*ARID1B*	22	0.211
HPMRS1	239300	*PIGV*	21	0.250
KBGS	148050	*ANKRD11*	20	0.195
GestaltMatcher				
DEE66	618067	*PACS2*	33	0.358
DeepGestalt and GestaltMatcher				
SHMS	615009	*PACS1*	37	0.467
DEE66	618067	*PACS2*	30	0.366
VRJS	615583	*PUF60*	26	0.357
NOCGUS	618652	*WDR37*	23	0.363
BRWS1	243310	*ACTB*	20	0.359

SHMS, Schuurs–Hoeijmakers syndrome; BRWS1, Baraitser–Winter syndrome 1; NS1, Noonan syndrome 1; KABUK1, Kabuki syndrome 1; RSTS1, Rubinstein–Taybi syndrome 1; AS, Angelman syndrome; NF1, Neurofibromatosis, type 1; CdLS, Cornelia de Lange syndrome; CSS, Coffin–Siris syndrome; HPMRS1, Hyperphosphatasia with impaired intellectual development syndrome 1; KBGS, KBG syndrome; DEE66, Developmental and Epileptic Encephalopathy 66; VRJS, Verheij syndrome; NOCGUS, Neuro-Oculo-Cardio-Genitourinary syndrome; #, number.

## Data Availability

The raw data supporting the conclusions of this article will be made available by the authors on request.

## Supplementary Materials

The following supporting information can be downloaded at https://www.mdpi.com/article/10.3390/ijms26167964/s1.

## Abbreviations

The following abbreviations are used in this manuscript:

AI	Artificial intelligence
BRWS1	Baraitser–Winter syndrome 1
CdLS	Cornelia de Lange syndrome
CEICA	Ethics and clinical research committee of the Government of Aragon
CNS	Central nervous system
DEE66	Developmental and Epileptic Encephalopathy-66
F2G	Face2Gene
HDAC	Histone deacetylase
HPO	Human phenotype ontology
NDD	Neurodevelopmental disorder
NOCGUS	Neuro-Oculo-Cardio-Genitourinary syndrome
OMIM	Online Mendelian Inheritance in Man
RGD	Rare genetic disorder
SHMS	Schuurs–Hoeijmakers syndrome
PPI	Protein–protein interaction
VRJS	Verheij syndrome
