# Supplementary material for: AI-Based Facial Phenotyping Supports a Shared Molecular Axis in PACS1-, PACS2-, and WDR37-Related Syndromes
Source: Int J Mol Sci. 2025 Aug 18;26(16):7964. doi: 10.3390/ijms26167964 (PMC12386744; doi:10.3390/ijms26167964)
Supplement: Supplementary file 1 [file ijms-26-07964-s001.zip › ijms-3734220-supplementary.pdf]

Table S1: Schuurs-Hoeijmakers syndrome (SHMS) identification using the DeepGestalt algorithm (v.22.3.0).

Patient ID	Sex	Age Range	*Gestalt* Score	Position
I-1	M	1y-3y	0,30	2
I-2	M	1y-3y	0,47	1
I-3	M	1y-3y	0,46	1
I-4	F	3y-6y	0,31	1
I-5	M	3y-6y	0,11	3
I-6	M	3y-6y	0,30	1
I-7	M	6y-12y	0,30	3
I-8	F	6y-12y	0,68	1
I-9	F	12y-18y	0,11	6
I-10	M	12y-18y	n/a	n/a
I-11	M	12y-18y	0,17	7
I-12	M	18y-40y	0,30	1
I-13	M	18y-40y	0,07	3
I-14	F	18y-40y	0,27	2

M, male; F, female; y, year; n/a, not available.

Table S2: Individuals with Schuurs-Hoeijmakers syndrome (SHMS) published in the literature whose photographs have been use in this study.

Patient ID	Sex	Age Range	Reference
R1	M	3y-6y	(1)
R2	M	12y-18y	(1)
R3	M	1y-3y	(2)
R5	F	1y-3y	(3)
R6	M	1y-3y	(3)
R7	M	6y-12y	(4)
R8	M	3y-6y	(5)
R9	F	3y-6y	(6)
R10	F	3y-6y	(6)
R11	M	3y-6y	(6)
R12	M	12y-18y	(7)
R13	F	6y-12y	(7)
R14	M	3y-6y	(7)
R15	M	3y-6y	(7)
R16	M	3y-6y	(7)
R17	M	18y-40y	(7)
R18	M	6y-12y	(7)
R19	M	6y-12y	(7)
R20	M	1y-3y	(7)
R21	F	3y-6y	(7)
R23	M	1y-3y	(7)
R24	M	3y-6y	(7)
R25	F	18y-40y	(7)
R26	F	18y-40y	(6)

M, male; F, female; y, year.

Table S3: Individuals with Developmental and epileptic encephalopathy 66 (DEE66), Neuro-oculo-cardio-genitourinary syndrome (NOCGUS), Verheij syndrome (VRJS), and Baraitser-Winter syndrome 1 (BRWS1) published in the literature, whose photographs have been used in this study.

Patient ID	Syndrome_ID	OMIM_ID	Sex	Age Range	Reference
P1	DEE66	618067	M	1y-3y	(8)
P2	DEE66	618067	M	1y-3y	(9)
P3	DEE66	618067	F	1y-3y	(10)
P4	DEE66	618067	F	3y-6y	(11)
P5	DEE66	618067	F	3y-6y	(8)
P6	DEE66	618067	M	6y-12y	(8)
P7	DEE66	618067	M	6y-12y	(12)
P8	DEE66	618067	M	6y-12y	(8)
P9	DEE66	618067	M	12y-18y	(8)
P10	DEE66	618067	F	12y-18y	(8)
P11	DEE66	618067	M	18y-40y	(13)
B1	BRWS1	243310	M	3y-6y	(14)
B2	BRWS1	243310	M	1y-3y	(14)
B3	BRWS1	243310	M	6y-12y	(15)
B4	BRWS1	243310	M	3y-6y	(15)
B5	BRWS1	243310	M	3y-6y	(15)
B6	BRWS1	243310	M	1y-3y	(16)
B7	BRWS1	243310	M	3y-6y	(16)
B8	BRWS1	243310	F	18y-40y	(17)
B9	BRWS1	243310	M	12y-18y	(17)
B10	BRWS1	243310	F	3y-6y	(18)
B11	BRWS1	243310	F	18y-40y	(18)
B12	BRWS1	243310	F	12y-18y	(18)
B13	BRWS1	243310	M	6y-12y	(18)
B14	BRWS1	243310	M	6y-12y	(18)
B15	BRWS1	243310	M	1y-3y	(18)
B16	BRWS1	243310	M	1y-3y	(18)
B17	BRWS1	243310	M	1y-3y	(18)
B18	BRWS1	243310	F	1y-3y	(18)
B19	BRWS1	243310	M	na	(19)
B20	BRWS1	243310	M	na	(19)
B21	BRWS1	243310	F	na	(19)
B22	BRWS1	243310	F	na	(19)
B23	BRWS1	243310	M	na	(19)
B24	BRWS1	243310	M	na	(19)
B25	BRWS1	243310	F	na	(19)
B26	BRWS1	243310	M	na	(19)
B27	BRWS1	243310	F	na	(19)
B28	BRWS1	243310	M	na	(19)
B29	BRWS1	243310	M	na	(19)
B30	BRWS1	243310	F	na	(19)
B31	BRWS1	243310	M	na	(19)
B32	BRWS1	243310	F	na	(19)
V1	VRJS	615583	F	1y-3y	(20)
V2	VRJS	615583	M	3y-6y	(20)

V3	VRJS	615583	M	12y-18y	(20)
V4	VRJS	615583	F	6y-12y	(20)
V5	VRJS	615583	F	12y-18y	(21)
V6	VRJS	615583	M	6y-12y	(22)
V7	VRJS	615583	M	6y-12y	(22)
V8	VRJS	615583	F	3y-6y	(22)
V9	VRJS	615583	F	6y-12y	(22)
V10	VRJS	615583	F	12y-18y	(22)
V11	VRJS	615583	F	1y-3y	(22)
V12	VRJS	615583	F	6y-12y	(22)
V13	VRJS	615583	M	6y-12y	(22)
V14	VRJS	615583	F	3y-6y	(22)
V15	VRJS	615583	F	1y-3y	(23)
V16	VRJS	615583	M	1y-3y	(23)
V17	VRJS	615583	F	>40y	(23)
V18	VRJS	615583	F	6y-12y	(23)
V19	VRJS	615583	F	12y-18y	(23)
V20	VRJS	615583	F	6y-12y	(23)
V21	VRJS	615583	M	18y-40y	(23)
V22	VRJS	615583	M	3y-6y	(23)
V23	VRJS	615583	F	3y-6y	(22)
W1	NOCGUS	618652	M	1y-3y	(24)
W2	NOCGUS	618652	F	1y-3y	(25)
W3	NOCGUS	618652	F	3y-6y	(26)
W4	NOCGUS	618652	F	3y-6y	(25)
W5	NOCGUS	618652	M	1y-3y	(25)
W6	NOCGUS	618652	M	6y-12y	(24)
W7	NOCGUS	618652	M	12y-18y	(26)
W8	NOCGUS	618652	M	12y-18y	(26)
W9	NOCGUS	618652	F	18y-40y	(25)
W10	NOCGUS	618652	M	18y-40y	(24)

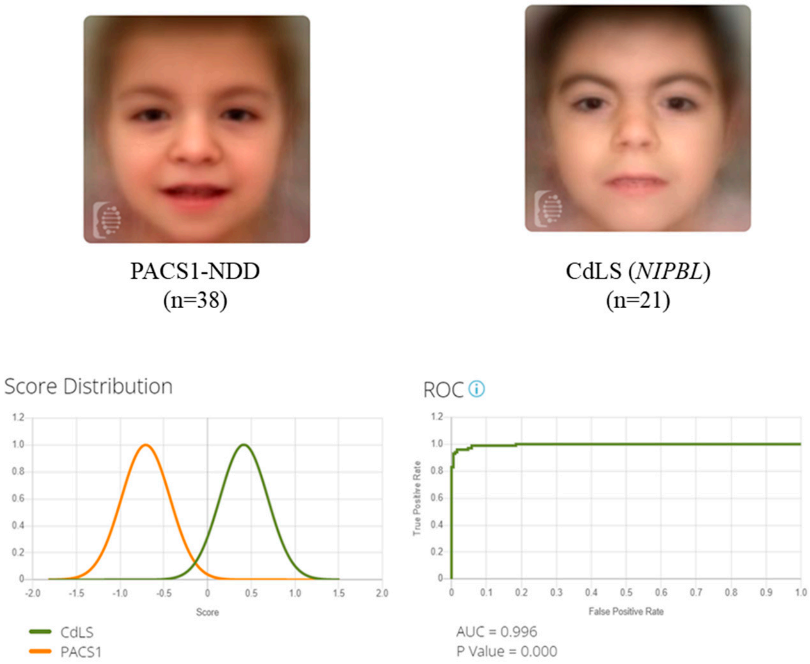

Figure S1: Binary comparison of facial images from individuals with SHMS (n=38) and CdLS (n=21). Top: Composite gestalt image generated from the analyzed photographs. Bottom: Score distribution and receiver operating characteristic (ROC) curve obtained through GestaltMatcher analysis.

References

1. Schuurs-Hoeijmakers JHM, Oh EC, Vissers LELM, Swinkels MEM, Gilissen C, Willemsen MA, et al. Recurrent de novo mutations in PACS1 cause defective cranial-neural-crest migration and define a recognizable intellectual-disability syndrome. Am J Hum Genet. 2012 Dec 7;91(6):1122–7.

2. Gadzicki D, Döcker D, Schubach M, Menzel M, Schmorl B, Stellmer F, et al. Expanding the phenotype of a recurrent de novo variant in PACS1 causing intellectual disability. Clin Genet. 2015 Sep 1;88(3):300–2.

3. Hoshino Y, Enokizono T, Imagawa K, Tanaka R, Suzuki H, Fukushima H, et al. Schuurs-Hoeijmakers syndrome in two patients from Japan. Am J Med Genet A. 2019 Mar ;179(3):341–3.

4. Kurt Colak F, Eyerci N, Aytekin C, Eksioglu AS. Renpenning Syndrome in a Turkish Patient: de novo Variant c.607C>T in PACS1 and Hypogammaglobulinemia Phenotype. Mol Syndromol. 2020 Jul 1;11(3):157–61.

5. Abdulqader SA, Wli WA, Qaryaqos SH. Schuurs-Hoeijmakers syndrome in a patient from Iraq - Kirkuk. Clin case reports. 2021 Oct 1;9(10).

6. Tenorio-Castaño J, Morte B, Nevado J, Martinez-Glez V, Santos-Simarro F, García-Miñaúr S, et al. Schuurs-Hoeijmakers Syndrome (PACS1 Neurodevelopmental Disorder): Seven Novel Patients and a Review. Genes (Basel). 2021;12(5).

7. Schuurs-Hoeijmakers JHM, Landsverk ML, Foulds N, Kukolich MK, Gavrilova RH, Greville-Heygate S, et al. Clinical delineation of the PACS1-related syndrome--Report on 19 patients. Am J Med Genet A. 2016 Mar 1;170(3):670–5.

8. Olson HE, Jean-Marçais N, Yang E, Heron D, Tatton-Brown K, van der Zwaag PA, et al. A Recurrent De Novo PACS2 Heterozygous Missense Variant Causes Neonatal-Onset Developmental Epileptic Encephalopathy, Facial Dysmorphism, and Cerebellar Dysgenesis. Am J Hum Genet. 2018;102(5):995–1007.

9. Cesaroni E, Matricardi S, Cappanera S, Marini C. First reported case of an inherited PACS2 pathogenic variant with variable expression. Epileptic Disord. 2022;24(3):572–6.

10. Terrone G, Marchese F, Vari MS, Severino M, Madia F, Amadori E, et al. A further contribution to the delineation of epileptic phenotype in PACS2-related syndrome. Seizure. 2020;79:53–5.

11. Sánchez-Soler MJ, Serrano-Antón AT, López-González V, Guillén-Navarro E. New case with the recurrent c.635G>A pathogenic variant in the PACS2 gene: Expanding the phenotype. Neurologia. 2021;36(9):716–9.

12. Dentici ML, Barresi S, Niceta M, Ciolfi A, Trivisano M, Bartuli A, et al. Expanding the clinical spectrum associated with PACS2 mutations. Clin Genet. 2019;95(4):525–31.

13. Sakaguchi Y, Yoshihashi H, Uehara T, Miyama S, Kosaki K, Takenouchi T. Coloboma may be a shared feature in a spectrum of disorders caused by mutations in the WDR37-PACS1-PACS2 axis. Am J Med Genet A. 2021;185(3):884–8. /

14. Nie K, Huang J, Liu L, Lv H, Chen D, Fan W. Identification of a De Novo Heterozygous Missense ACTB Variant in Baraitser-Winter Cerebrofrontofacial Syndrome. Front Genet. 2022;13.

15. Baumann M, Beaver EM, Palomares-Bralo M, Santos-Simarro F, Holzer P, Povysil G, et al. Further delineation of putative ACTB loss-of-function variants: A 4-patient series. Hum Mutat. 2020;41(4):753–8.

16. Chacon-Camacho OF, Barragán-Arévalo T, Villarroel CE, Almanza-Monterrubio M, Zenteno JC. Previously undescribed phenotypic findings and novel ACTG1 gene pathogenic variants in Baraitser-Winter cerebrofrontofacial syndrome. Eur J Med Genet. 2020;63(5).

17. Hampshire K, Martin PM, Carlston C, Slavotinek A. Baraitser-Winter cerebrofrontofacial syndrome: Report of two adult siblings. Am J Med Genet A. 2020;182(8):1923–32.

18. Donato N Di, Kuechler A, Vergano S, Heinritz W, Bodurtha J, Merchant SR, et al. Update on the ACTG1-associated Baraitser-Winter cerebrofrontofacial syndrome. Am J Med Genet A. 2016;170(10):2644–51.

19. Verloes A, Donato N Di, Masliah-Planchon J, Jongmans M, Abdul-Raman OA, Albrecht B, et al. Baraitser-Winter cerebrofrontofacial syndrome: delineation of the spectrum in 42 cases. Eur J Hum Genet. 2015;23(3):292–301.

20. Chehadeh S El, Kerstjens-Frederikse WS, Thevenon J, Kuentz P, Bruel AL, Thauvin-Robinet C, et al. Dominant variants in the splicing factor PUF60 cause a recognizable syndrome with intellectual disability, heart defects and short stature. Eur J Hum Genet [Internet]. 2016;25(1):43–51.

21. Yamada M, Uehara T, Suzuki H, Takenouchi T, Kosaki K. Protein elongation variant of PUF60: Milder phenotypic end of the Verheij syndrome. Am J Med Genet A. 2020;182(11):2709–14.

22. Low KJ, Ansari M, Jamra RA, Clarke A, Chehadeh S El, Fitzpatrick DR, et al. PUF60 variants cause a syndrome of ID, short stature, microcephaly, coloboma, craniofacial, cardiac, renal and spinal features. Eur J Hum Genet. 2017;25(5):552–9.

23. Fennell AP, Baxter AE, Berkovic SF, Ellaway CJ, Forwood C, Hildebrand MS, et al. The diverse pleiotropic effects of spliceosomal protein PUF60: A case series of Verheij syndrome. Am J Med Genet A. 2022;188(12):3432–47.

24. Reis LM, Sorokina EA, Thompson S, Muheisen S, Velinov M, Zamora C, et al. De Novo Missense Variants in WDR37 Cause a Severe Multisystemic Syndrome. Am J Hum Genet. 2019;105(2):425–33.

25. Kanca O, Andrews JC, Lee PT, Patel C, Braddock SR, Slavotinek AM, et al. De Novo Variants in WDR37 Are Associated with Epilepsy, Colobomas, Dysmorphism, Developmental Delay, Intellectual Disability, and Cerebellar Hypoplasia. Am J Hum Genet. 2019;105(2):413–24.

26. Hay E, Henderson RH, Mansour S, Deshpande C, Jones R, Nutan S, et al. Expanding the phenotypic spectrum consequent upon de novo WDR37 missense variants. Clin Genet. 2020 Aug 1;98(2):191–7.
